# On the Nuisance Parameter Elimination Principle in Hypothesis Testing

**DOI:** 10.3390/e26020117

**Published:** 2024-01-29

**Authors:** Andrés Felipe Flórez Rivera, Luis Gustavo Esteves, Victor Fossaluza, Carlos Alberto de Bragança Pereira

**Affiliations:** Institute of Mathematics and Statistics, University of São Paulo, São Paulo 05508-090, Brazil; lesteves@ime.usp.br (L.G.E.); victorf@ime.usp.br (V.F.); cpereira@ime.usp.br (C.A.d.B.P.)

**Keywords:** *p*-values, Bayes factor, likelihood function, hypothesis testing

## Abstract

The Non-Informative Nuisance Parameter Principle concerns the problem of how inferences about a parameter of interest should be made in the presence of nuisance parameters. The principle is examined in the context of the hypothesis testing problem. We prove that the mixed test obeys the principle for discrete sample spaces. We also show how adherence of the mixed test to the principle can make performance of the test much easier. These findings are illustrated with new solutions to well-known problems of testing hypotheses for count data.

## 1. Introduction

Principles of Statistical Inference (or Data Reduction) constitute important guidelines on how to draw conclusions from data, especially when performing standard inferential procedures for unknown parameters of interest, like estimation and hypothesis testing. For instance, the Sufficiency Principle (SP) states that any sufficient statistic retains all relevant information about the unknown parameters that should be used to make inferences about them. It precisely recommends that if *T* is a sufficient statistic for the statistical model under consideration and $x_{1}$ and $x_{2}$ are sample points such that $T\left(x_{1}\right)=T\left(x_{2}\right)$, then the observation of any of these points should lead to the same conclusions regarding the parameters of interest.

Besides the place of sufficiency in Statistical Inference, these recommendations cover several issues such as the contrast between post-experimental and pre-experimental reasoning and the roles of non-informative stopping rules, censoring mechanisms and nuisance parameters in data analysis. Among the main principles, the Sufficiency Principle is generally recognized as a cornerstone of Statistical Inference. On the other hand, the Likelihood Principle (LP) and its profound consequences are still subjects of intense debate. The reader will find a detailed discussion of the Likelihood Principle in [1,2,3,4,5,6].

In this work, we examine the Non-Informative Nuisance Parameter Principle (NNPP) introduced by Berger and Wolpert in 1988 in their remarkable book that concerns the problem of the way inferences about a parameter of interest should be made in the presence of nuisance parameters. Nuisance parameters usually affect inferences about the parameter of interest, like in the estimation of the mean of a normal distribution with unknown variance, in the estimation of the parameters of a linear regression model in the presence of unknown variance, and in the determination of *p*-values for specific hypotheses in the analysis of 2×2 contingency tables ([7]). In a few words, the NNPP states that under suitable conditions, it is irrelevant whether the value of a non-informative nuisance parameter is known or not in order to draw conclusions about the parameter of interest. Despite the importance of the problem for eliminating nuisance parameters in data analysis, the authors have not explored this principle and its consequences in some depth as far as we have reviewed the literature. For this reason, we revisit the NNPP by formally stating it for the problem of hypothesis testing, present decision rules that meet the principle and show how the performance of a particular test in line with the NNPP can then be simplified.

This work is organized as follows: in Section 2, the NNPP for hypothesis testing is stated, discussed and illustrated under a Bayesian perspective. In Section 3, the Bayesian test procedure based on the concept of adaptive significance level and on an alternative *p*-value introduced by Pericchi and Pereira in [8], henceforth named the mixed test, is reviewed and is proven to satisfy the NNPP for discrete sample data when the (marginal) null hypothesis regarding the parameter of interest is a singleton (as a matter of fact, the result also holds when such a null hypothesis is specified by a hyperplane). In that section, we also define conditional versions of the adaptive significance level and *p*-value based on suitable statistics and prove that under those conditions, the performance of the mixed test is then simply the comparison between these new conditional quantities. These results are of great importance to make it easier to use the mixed test in various situations. In Section 4, we exemplify the main results by presenting new solutions by using the mixed test for well-known problems of test of hypotheses for count data under suitable reparametrizations of the corresponding models: we revisit the problems of comparison of Poisson population means and of testing the hypotheses of independence and symmetry in contingency tables. We make our final comments in Section 5. The proofs of the theorems and the calculations for one example in Section 4 are found in the Appendix A.

## 2. The Non-Informative Nuisance Parameter Principle for Hypothesis Testing

The problem of the elimination of nuisance parameters in statistical inference has a long history and remains a major issue. Proposals to deal with it include the marginalization of the likelihood function by integrating out the nuisance parameter ([9,10,11]), the construction of partial likelihood functions ([12,13,14], among others) and the consideration of conditional likelihood functions based on different notions of non-informativeness, sufficiency and ancillarity. Elimination of nuisance parameters and different notions of non-information have also been studied in more detail in [15,16,17,18], where, based on suitable statistics, the concepts of B, S and G non-information are presented. The generalized Sufficiency and Conditionality Principles are also discussed in [17]. On the other hand, Bayesian methods for eliminating nuisance parameters based on a suitable statistic *T* involve different definitions of sufficiency: for instance, K-Sufficiency, Q-Sufficiency and L-Sufficiency (see for example [17] and references therein).

In this section, the Non-Informative Nuisance Parameter Principle (NNPP) by Berger and Wolpert is discussed and formally defined for the problem of hypothesis testing. As we will see, on the one hand, the NNPP seems to be fair under both the partial and the conditional non-Bayesian approaches mentioned in the previous paragraph; on the other hand, it sounds really reasonable under the Bayesian standpoint. Despite the relevance of the problem of the elimination of nuisance parameters in data analysis, Berger and Wolpert [1] presented the NNPP but has not explored the principle in-depth as far as we have examined in the literature.

Some notation is needed to continue. We denote by θ the unknown parameter and by *X* the sample to be observed. Θ and X represent the parameter and the sample spaces, respectively. The family of discrete probability distributions for *X* is denoted by P={P(·|θ):θ∈Θ}. In addition, for x∈X, $L_{x}\left(\cdot \right)$ denotes the likelihood function for θ generated by the sample point *x*. By an experiment E, we mean, as in [1], a triplet E=(X,θ,P), with *X*, θ and P as defined earlier. Finally, for a subset $\Theta_{0}$ of Θ, we formulate the null hypothesis $H:\theta \in \Theta_{0}$ and the alternative one $A:\theta \notin \Theta_{0}$. We recall that a test function (procedure) for the hypotheses *H* versus *A* is a function φ:X→{0,1} that takes the value 1 (φ(x)=1) if *H* is rejected when x∈X is observed and takes the value 0 (φ(x)=0) if *H* is not rejected when *x* is observed. Under the Bayesian perspective, we also consider a continuous prior density function π(·) for θ that induces, when combined with the likelihood function $L_{x}\left(\cdot \right)$, a continuous posterior density function for θ given *x*, π(·|x).

In [1], Berger and Wolpert presented the following principle on how to make inferences about an unknown parameter of interest $\theta_{1}$ in the presence of a nuisance parameter $\theta_{2}$: when a sample observation, say $x_{0}$, separates information concerning $\theta_{1}$ from information on $\theta_{2}$, it is irrelevant whether the value of $\theta_{2}$ is known or unknown in order to make inferences about $\theta_{1}$ based on the observation of $x_{0}$. In other terms, if the conclusions on $\theta_{1}$ were to be the same for every possible value of the nuisance parameter, were $\theta_{2}$ known, then the same conclusions on $\theta_{1}$ should be reached even if $\theta_{2}$ is unknown. These authors then consider the following mathematical setup to formalize these ideas.

Let $\theta =(\theta_{1},\theta_{2})$, with $\theta_{1}$ and $\theta_{2}$ defined as in the previous paragraph. Consider $\Theta =\Theta_{1}\times \Theta_{2}$; that is, the parameter space is variation independent, where $\Theta_{i}\subseteq R^{n_{i}}$ is the set of values for $\theta_{i}$, $n_{i}\in N^{*}$, i=1,2. Suppose the experiment E=(X,θ,P) is carried out to learn about θ. Let $\bar{E}=\left(\left(X,\theta_{2}\right),\theta_{1},\bar{P}\right)$ be the “thought” experiment in which the pair $\left(X,\theta_{2}\right)$ is to be observed (instead of observing only *X*), where $\bar{P}$ is the family of distributions for $\left(X,\theta_{2}\right)$ indexed by $\theta_{1}$. Suppose also that under experiment E, the likelihood function generated by a specific $x_{0}\in X$ for θ has the following factored form:

(1) $$L_{x_{0}}\left(\theta_{1},\theta_{2}\right)\;=\;L_{x_{0}}^{1}\left(\theta_{1}\right)L_{x_{0}}^{2}\left(\theta_{2}\right)$$

where $L_{x_{0}}^{i}:\Theta_{i}\to R_{+}$, i=1,2; that is, $L_{x_{0}}^{i}$ depends on θ only through $\theta_{i}$.

Berger and Wolpert then states the **Non-Informative Nuisance Parameter Principle (NNPP)**: *if E and $x_{0}\in X$ are such that (1) holds, and if the inference about $\theta_{1}$ from the observation of $\left(x_{0},\theta_{2}\right)$ when $\bar{E}$ is performed does not depend on $\theta_{2}$, then the inferential statements made for $\theta_{1}$ from E and $x_{0}$ should be the same as (should coincide with) the inferential statements made from $\bar{E}$ and $\left(x_{0},\theta_{2}\right)$ for every $\theta_{2}\in \Theta_{2}$*.

The authors named such a parameter $\theta_{2}$ a **Non-Informative Nuisance Parameter (NNP)**, as the conclusions or decisions regarding $\theta_{1}$ from $\bar{E}$ and $\left(x_{0},\theta_{2}\right)$ do not depend on $\theta_{2}$.

A likelihood function that satisfies (1) is named a likelihood function with separable parameters ([19]). The factored form of the likelihood function in (1) seems to capture the notion of “absence of information about a parameter, say $\theta_{1}$, from the other, $\theta_{2}$, and vice versa” under both Bayesian and non-Bayesian reasoning. Indeed, under the Bayesian paradigm, posterior independence between $\theta_{1}$ and $\theta_{2}$ (say, given $x_{0}$) reflects the fact that one´s opinion about the parameter $\theta_{1}$ after observing $x_{0}$ is not altered by any information about $\theta_{2}$, and consequently, decisions regarding $\theta_{1}$ should not depend on $\theta_{2}$. Since posterior independence between $\theta_{1}$ and $\theta_{2}$ given $x_{0}$ is equivalent to the factored form of the likelihood function generated by $x_{0}$ under prior independence, condition (1) sounds really reasonable as a mathematical description of separate information about the parameters. Thus, if a Bayesian statistician should make inferences regarding a parameter $\theta_{1}$ in the presence of a nuisance parameter $\theta_{2}$, it would be ideal that these parameters are independent a posteriori; that is, the factored form of the likelihood function holds. This last equivalence is proven in the theorem below.

**Theorem** **1.** *Let E=(X,θ,P) be an experiment and π(·) be the prior probability density function for $\theta =(\theta_{1},\theta_{2})$. Suppose $\theta_{1}$ is independent of $\theta_{2}$ ($\theta_{1}⫫\theta_{2}$) a priori. Then, for each x∈X*,

(2) $$\theta_{1}⫫\theta_{2}\;|\;X=x\;⟺\;\exists L_{x}^{i}:\Theta_{i}\to R_{+}\;,\;i=1,2,$$

*such that $L_{x}\left(\theta_{1},\theta_{2}\right)=L_{x}^{1}\left(\theta_{1}\right)L_{x}^{2}\left(\theta_{2}\right)$*.

On the other hand, the condition (1) seems to also be a fair representation of non-informativeness of one parameter on another under a non-Bayesian perspective. In fact, such a factored form of the likelihood function arises, for instance, when the sample *X* is conditioned on particular types of statistics that are simple to interpret under non-Bayesian paradigms. Note that for any statistic *T*, one can write

$$L_{x}\left(\theta_{1},\theta_{2}\right)=P\left(X=x|\theta_{1},\theta_{2}\right)=P\left(X=x|T\left(X\right)=T\left(x\right),\theta_{1},\theta_{2}\right)P\left(T\left(X\right)=T\left(x\right)|\theta_{1},\theta_{2}\right)\;.$$

If, in addition, *T* is a statistic such that its distribution given θ depends only on $\theta_{1}$ and the conditional distribution of *X* given T(X)=T(x), and θ depends only on $\theta_{2}$, the factored form in (1) is easily obtained (such a statistic was named p-sufficient for $\theta_{1}$ by Basu ([17]). In this situation, all the relevant information on $\theta_{1}$ is summarized in *T*, and one can fully make inferences on $\theta_{1}$ taking into account only the conditional distribution of *T* given θ, which does not involve $\theta_{2}$. Similarly, if *T* is a statistic such that its distribution given θ depends only on $\theta_{2}$ and the conditional distribution of *X* given T(X)=T(x) and θ depends only on $\theta_{1}$, the factored form in (1) holds. Such a statistic was named s-ancillary for $\theta_{1}$ by Basu ([17]), and it is somewhat evident that in this case, conclusions on $\theta_{1}$ should be drawn exclusively from the distribution of *X* given T(X) and θ, which does not depend on $\theta_{2}$. Such a conditional approach to the problem of elimination of nuisance parameters had already been proposed by Basu ([17]) and in a sense is closely related to the NNPP by Berger and Wolpert. The next theorem formally presents such results.

**Theorem** **2.** *Let E=(X,θ,P) be an experiment in which $\theta =(\theta_{1},\theta_{2})$ and Θ is variation independent. Then, if ∃T:X→T such that T is either p-sufficient or s-ancillary for $\theta_{1}$, then for each x∈X, the likelihood function generated by x, $L_{x}\left(\cdot \right)$ can be factored as (1)*.

In summary, it seems reasonable that inferences about $\theta_{1}$ and $\theta_{2}$ can be performed independently under condition (1). Thus, if only $\theta_{1}$ is of interest, then it seems sensible under (1) that we reach the same conclusions on $\theta_{1}$ when *x* is observed either by using the whole likelihood function $L_{x}$ or only the factor $L_{x}^{1}$. That is, it makes sense to disregard the information contained in $L_{x}^{2}$ and focus on $L_{x}^{1}$. As mentioned by [19], examples of likelihood functions with separable parameters like (1) are rare, but if (1) holds, it would be a useful property for Bayesian and non-Bayesian statisticians to analyze statistical data, especially in the presence of nuisance parameters. This fact will be illustrated in Section 3 and Section 4.

We end this section by formally adapting the general NNPP to the special problem of hypothesis testing, in which inference about an unknown parameter consists of deciding whether a statement about the parameter (a statistical hypothesis) should be rejected or accepted by using the observable quantity *X*.

As before, let E=(X,θ,P) be an experiment, with $\Theta =\Theta_{1}\times \Theta_{2}$. Let $\bar{E}=\left(\left(X,\theta_{2}\right),\theta_{1},\bar{P}\right)$ be the “thought” experiment in which, in addition to *X*, $\theta_{2}$ is observed. Then, consider the following definition.

**Definition** **1.** ***Non-Informative Nuisance Parameter (NNP)**: Let $B\subseteq \Theta_{1}$ and $\bar{\varphi }:X\times \Theta_{2}\to \left\{0,1\right\}$ be a test for the hypotheses*

(3) $$\begin{array}{cc} \bar{H}: & \;\theta_{1}\in B\\ \bar{A}: & \;\theta_{1}\notin B \end{array}$$

*Then, we say that $\theta_{2}$ is a Non-Informative Nuisance Parameter (NNP) for testing $\bar{H}$ versus $\bar{A}$ by using $\bar{\varphi }$ if, for every x∈X such that (1) holds, $\bar{\varphi }\left(x,\theta_{2}\right)$ does not depend on $\theta_{2}$; that is, it depends only on x*.

In a nutshell, Definition 1 tells us something that appears intuitive: if the decision between *H* and *A* does not depend on $\theta_{2}$, then $\theta_{2}$ does not provide any information about $\theta_{1}$. In the following example, we illustrate this idea.

**Example** **1.** *Consider that $\Theta_{1}=\Theta_{2}=R$ and the experiment $\bar{E}=\left(\left(X,\theta_{2}\right),\theta_{1},P\right)$. Let B⊂R and $\bar{\varphi }:X\times \Theta_{2}\to \left\{0,1\right\}$ be the test for the hypotheses*

(4) $$\begin{array}{cc} \bar{H}: & \;\theta_{1}\in B\\ \bar{A}: & \;\theta_{1}\notin B, \end{array}$$

*such that the null hypothesis is rejected when the conditional probability of B given x and $\theta_{2}$ is small; that is*,

(5) $$\bar{\varphi }\left(x,\theta_{2}\right)=1\Leftrightarrow P\left(\theta_{1}\in B\;|\;x,\theta_{2}\right)<\delta ,$$

*where δ∈(0,1). Suppose, in addition, that $\theta_{1}$ and $\theta_{2}$ are independent a priori. Let us verify that $\theta_{2}$ is an NNP for testing these hypotheses by means of $\bar{\varphi }$. Let $x_{0}\in X$ be such that $L_{x_{0}}\left(\theta_{1},\theta_{2}\right)=L_{x_{0}}^{1}\left(\theta_{1}\right)L_{x_{0}}^{2}\left(\theta_{2}\right)$ for specific functions $L_{x_{0}}^{1}$ and $L_{x_{0}}^{2}$. Then*,

(6) $$\begin{array}{cc} P(\theta_{1}\in B\;|\;x_{0},\theta_{2})= & \int_{B}\pi \left(\theta_{1}\;|\;x_{0},\theta_{2}\right)d\theta_{1}=\\ = & \int_{B}\left[\frac{P\left(X=x_{0}|\theta_{1},\theta_{2}\right)\pi \left(\theta_{2}|\theta_{1}\right)\pi_{1}\left(\theta_{1}\right)}{\int_{\Theta_{1}}P\left(X=x_{0}|\theta_{1}^{\prime },\theta_{2}\right)\pi \left(\theta_{2}|\theta_{1}^{\prime }\right)\pi_{1}\left(\theta_{1}^{\prime }\right)d\theta_{1}^{\prime }}\right]d\theta_{1}\;=\\ = & \frac{\int_{B}L_{x_{0}}\left(\theta_{1},\theta_{2}\right)\pi_{2}\left(\theta_{2}\right)\pi_{1}\left(\theta_{1}\right)d\theta_{1}}{\int_{\Theta_{1}}L_{x_{0}}\left(\theta_{1},\theta_{2}\right)\pi_{2}\left(\theta_{2}\right)\pi_{1}\left(\theta_{1}\right)d\theta_{1}}\;=\\ = & \frac{\int_{B}L_{x_{0}}^{1}\left(\theta_{1}\right)L_{x_{0}}^{2}\left(\theta_{2}\right)\pi_{2}\left(\theta_{2}\right)\pi_{1}\left(\theta_{1}\right)d\theta_{1}}{\int_{\Theta_{1}}L_{x_{0}}^{1}\left(\theta_{1}\right)L_{x_{0}}^{2}\left(\theta_{2}\right)\pi_{2}\left(\theta_{2}\right)\pi_{1}\left(\theta_{1}\right)d\theta_{1}}\;\Rightarrow \\ \Rightarrow & \;P\left(\theta_{1}\in B\;|\;x_{0},\theta_{2}\right)\;=\;\frac{\int_{B}L_{x_{0}}^{1}\left(\theta_{1}\right)\pi_{1}\left(\theta_{1}\right)d\theta_{1}}{\int_{\Theta_{1}}L_{x_{0}}^{1}\left(\theta_{1}\right)\pi_{1}\left(\theta_{1}\right)d\theta_{1}}\;, \end{array}$$

*where $\pi_{i}$ is the prior of $\theta_{i}$, i=1,2. Thus, we have that*

(7) $$\bar{\varphi }\left(x_{0},\theta_{2}\right)=1\;\Leftrightarrow \;\frac{\int_{B}L_{x_{0}}^{1}\left(\theta_{1}\right)\pi_{1}\left(\theta_{1}\right)d\theta_{1}}{\int_{\Theta_{1}}L_{x_{0}}^{1}\left(\theta_{1}\right)\pi_{1}\left(\theta_{1}\right)d\theta_{1}}\;<\;\delta .$$

*Note from Equation (7) that $\bar{\varphi }\left(x_{0},\theta_{2}\right)$ does not depend on $\theta_{2}$. Thus, $\theta_{2}$ is an NNP for testing $\bar{H}$ versus $\bar{A}$ by using $\bar{\varphi }$*.

After defining an NNP, we formally state the Non-Informative Nuisance Parameter Principle (NNPP) for hypothesis testing.

**Definition** **2.** ***Non-Informative Nuisance Parameter Principle (NNPP)**: Let the parameter space be variation independent; that is, $\Theta =\Theta_{1}\times \Theta_{2}$. Consider the experiments E=(X,θ,P) and $\bar{E}=\left(\left(X,\theta_{2}\right),\theta_{1},\bar{P}\right)$. Let $B\subseteq \Theta_{1}$ be the subset of $\Theta_{1}$ of interest. In addition, let φ:X→{0,1} and $\bar{\varphi }:X\times \Theta_{2}\to \left\{0,1\right\}$ be tests for the hypotheses*

(8) $$\begin{array}{cc} H: & \;\theta \in B\times \Theta_{2}\\ A: & \;\theta \notin B\times \Theta_{2}, \end{array}\;and\;\begin{array}{cc} \bar{H}: & \;\theta_{1}\in B\\ \bar{A}: & \;\theta_{1}\notin B, \end{array}$$

*respectively*.

*If $\theta_{2}$ is an NNP for testing $\bar{H}$ versus $\bar{A}$ by using $\bar{\varphi }$ and $x_{0}\in X$ such that condition (1) holds, then*

(9) $$\varphi \left(x_{0}\right)=1\;\Leftrightarrow \;\bar{\varphi }\left(x_{0},\theta_{2}\right)=1\;.$$

The NNPP for statistical hypothesis testing says that if one intends to test a hypothesis regarding only the parameter $\theta_{1}$, it is irrelevant whether $\theta_{2}$ is known or unknown if it is non-informative for such a decision-making problem. More formally, if one wants to test a hypothesis concerning only $\theta_{1}$ and he observes a sample point $x_{0}\in X$ that separates information on $\theta_{1}$ from information on $\theta_{2}$—that is, (1) holds—then the performances of the tests φ under the original experiment E and $\bar{\varphi }$ under the “thought” experiment $\bar{E}$ should yield the same decision on the hypothesis $\theta_{1}\in B$ if $\theta_{2}$ is non-informative for that purpose.

We should mention that the NNPP can be adapted to any other inferential procedure. However, in this work, we focus on the principle for the problem of hypothesis testing. We conclude this section by proving that tests based on the posterior probabilities of the hypotheses satisfy the NNPP under prior independence.

**Example** **2** (continuation of Example 1). *Consider the conditions of Example 1. Consider E=(X,θ,P) and let φ:X→{0,1} be the test for the hypotheses*

(10) $$\begin{array}{cc} H: & \;\theta \in B\times \Theta_{2}\\ A: & \;\theta \notin B\times \Theta_{2} \end{array}$$

*that rejects the null hypothesis H if its posterior probability is small; that is*,

(11) $$\varphi \left(x\right)=1\Leftrightarrow P\left(\theta \in B\times \Theta_{2}\;|\;x\right)<\delta .$$

*Let $x_{0}\in X$ be such that $L_{x_{0}}\left(\theta_{1},\theta_{2}\right)=L_{x_{0}}^{1}\left(\theta_{1}\right)L_{x_{0}}^{2}\left(\theta_{2}\right)$. We can write the posterior probability on the right-hand side of (11) as*

(12) $$\begin{array}{c} P\left(\theta \in B\times \Theta_{2}\;|\;x_{0}\right)\;=\;\int_{B\times \Theta_{2}}\pi \left(\theta \;|\;x_{0}\right)d\theta \;=\;\frac{\int_{B\times \Theta_{2}}L_{x_{0}}\left(\theta \right)\pi \left(\theta \right)d\theta }{\int_{\Theta }L_{x_{0}}\left(\theta \right)\pi \left(\theta \right)d\theta }\;=\;\\ =\frac{\int_{B\times \Theta_{2}}L_{x_{0}}^{1}\left(\theta_{1}\right)L_{x_{0}}^{2}\left(\theta_{2}\right)\pi_{1}\left(\theta_{1}\right)\pi_{2}\left(\theta_{2}\right)d\theta }{\int_{\Theta_{1}\times \Theta_{2}}L_{x_{0}}^{1}\left(\theta_{1}\right)L_{x_{0}}^{2}\left(\theta_{2}\right)\pi_{1}\left(\theta_{1}\right)\pi_{2}\left(\theta_{2}\right)d\theta }\;=\;\frac{\int_{B}L_{x_{0}}^{1}\left(\theta_{1}\right)\pi_{1}\left(\theta_{1}\right)d\theta_{1}}{\int_{\Theta_{1}}L_{x_{0}}^{1}\left(\theta_{1}\right)\pi_{1}\left(\theta_{1}\right)d\theta_{1}}\;, \end{array}$$

*where the last equality follows from Fubini’s Theorem. Hence*,

(13) $$\varphi \left(x\right)=1\Leftrightarrow \frac{\int_{B}L_{x_{0}}^{1}\left(\theta_{1}\right)\pi_{1}\left(\theta_{1}\right)d\theta_{1}}{\int_{\Theta_{1}}L_{x_{0}}^{1}\left(\theta_{1}\right)\pi_{1}\left(\theta_{1}\right)d\theta_{1}}<\delta .$$

From Equations (7) and (13), we have that $\varphi \left(x\right)=1\Leftrightarrow \bar{\varphi }\left(x,\theta_{2}\right)=1$. Thus, the NNP Principle is met by tests based on posterior probabilities, as in Example 1. This result also holds when $\Theta_{i}\subseteq R^{n_{i}}$, $n_{i}\in N^{*}$, i=1,2.

In the next section, we examine a second test procedure that is in line with the NNPP. We review the mixed test introduced by Pericchi and Pereira ([8]) and prove that such a test meets the NNPP for simple hypotheses concerning the parameter of interest. We also show how the adherence of the mixed test to the NNPP can then simplify its use.

## 3. The Mixed Test Procedure

The mixed test formally introduced in ([8]) is a test procedure that combines elements from both Bayesian and frequentist views. On the one hand, it considers an (intrinsically Bayesian) prior distribution for the parameter from which predictive distributions for the data under the competing hypotheses and Bayes factors are derived. On the other hand, the performance of the test depends on ordering the sample space by the Bayes factor and on the integration of these predictive distributions over specific subsets of the sample space in a frequentist-like manner. The mixed test is an optimal procedure in the sense that it minimizes linear combinations of averaged (weighted) probabilities of errors of decision. It also meets a few logical requirements for multiple-hypothesis testing and obeys the Likelihood Principle for discrete sample spaces despite the integration over the sample space it involves. In addition, the test overcomes several of the drawbacks fixed-level tests have. However, a difficulty with the mixed test procedure is the need to evaluate the Bayes factor for every sample point to order the sample space, which may involve intensive calculations. Properties of the mixed test and examples of application are examined in detail in [8,20,21,22,23,24,25].

Next, we review the general procedure for the performance of the mixed test and then show the test satisfies the NNPP when the hypothesis regarding the parameter of interest is a singleton.

First, we determine the predictive distributions for *X* under the competing hypotheses *H* and *A*, $f_{H}$ and $f_{A}$, respectively. For the null hypothesis $H:\theta \in \Theta_{0}$, $\Theta_{0}\subset \Theta$, $f_{H}$ is determined as follows: for each x∈X,

(14) $$f_{H}\left(x\right)\;=\;\int_{\Theta_{0}}L_{x}\left(\theta \right)dP_{H}\left(\theta \right)\;,$$

where $P_{H}$ denotes the conditional distribution of θ given $\theta \in \Theta_{0}$. That is, for each x∈X, $f_{H}\left(x\right)$ is the expected value of the likelihood function generated by *x* against $P_{H}$. Similarly, for the alternative hypothesis $A:\;\theta \in \Theta_{0}^{c}\;$ we define

(15) $$f_{A}\left(x\right)\;=\;\int_{\Theta_{0}^{c}}L_{x}\left(\theta \right)dP_{A}\left(\theta \right)\;,$$

where $P_{A}$ denotes the conditional distribution of θ given $\theta \in \Theta_{0}^{c}$. From (14) and (15), we obtain the Bayes factor of x∈X for the hypothesis *H* over *A* as

(16) $$BF\left(x\right)=\frac{f_{H}\left(x\right)}{f_{A}\left(x\right)}\;.$$

Finally, the mixed test $\varphi^{*}:\;X\to \left\{0,1\right\}$ for the hypotheses *H* versus *A* consists in rejecting *H* when x∈X is observed if and only if the Bayes factor BF(x) is small. That is, for each x∈X,

(17) $$\varphi^{*}\left(x\right)\;=\;1\;\;\Leftrightarrow \;\;BF\left(x\right)\;\leq \;b/a\;,$$

where the positive constants *a* and *b* reflect the decision maker’s evaluation of the impact of the errors of the two types or, equivalently, his prior preferences for the competing hypotheses. A detailed discussion on the specification of such constants is found in [8,20,21,22,23,24,25].

The mixed test can also be defined as a function of a new significance index. That is, (17) can be rewritten as a comparison between such a significance index and a specific cut-off value. These quantities are defined below.

For the mixed test defined in (17), the *p*-value of the observation $x_{0}\in X$ is the significance index given by

(18) $$p-value\left(x_{0}\right)\;=\;\sum_{x\;\in \;D(x_{0})}f_{H}\left(x\right)\;,$$

where $D\left(x_{0}\right)=\left\{x\in X:BF\left(x\right)\leq BF\left(x_{0}\right)\right\}$. Also, we define the adaptive type I error probability of $\varphi^{*}$ as

(19) $$\begin{array}{c} \alpha^{*}\;=\;\sum_{x\;\in \;D}f_{H}\left(x\right), \end{array}$$

where D={x∈X:BF(x)≤b/a}. Alternatively, $\alpha^{*}$ is also known as the adaptive significance level of $\varphi^{*}$.

Pereira et al. [21] proved that the mixed test $\varphi^{*}$ for the hypotheses

(20) $$\begin{array}{cc} H: & \theta \in \Theta_{0}\\ A: & \theta \in \Theta_{0}^{c} \end{array}$$

can be written as

(21) $$\varphi^{*}\left(x\right)\;=\;1\;\;\Leftrightarrow \;\;BF\left(x\right)\;\leq \;b/a\;\;\Leftrightarrow \;\;p-value\left(x\right)\;\leq \;\alpha^{*}\;.$$

Note that $\varphi^{*}$ consists of comparing the *p*-value with the cut-off $\alpha^{*}$, which depends on the specific statistical model under consideration and on the sample size, as opposed to a standard test with a fixed significance level that does not depend on the sample size.

The former does not have a few of the disadvantages of the latter, such as inconsistency ([8,26]) lack of correspondence between practical significance and statistical significance ([8,27]) and absence of logical coherence under multiple-hypothesis testing. We continue with the main results of the manuscript.

### The Mixed Test Obeys the NNPP

In this subsection, we prove that the mixed test meets the NNPP when the hypothesis about the parameter of interest is simple. Next, we examine further the case in which there is a statistic s-ancillary for the parameter of interest and show how the introduction of the concepts of a conditional *p*-value and a conditional adaptive significance level can make performance of the mixed test much easier.

**Theorem** **3.** Let $\theta =(\theta_{1},\theta_{2})$ and $\Theta =\Theta_{1}\times \Theta_{2}$ (that is, *Θ* is variation independent). Let E=(X,θ,P) and $\bar{E}=\left(\left(X,\theta_{2}\right),\theta_{1},\bar{P}\right)$ be two experiments as defined in Section 2. Let $\theta_{0}\in \Theta_{1}$. In addition, let $\varphi^{*}:X\to \left\{0,1\right\}$ and ${\bar{\varphi }}^{*}:X\times \Theta_{2}\to \left\{0,1\right\}$ be the mixed tests for the hypotheses

(22) $$\begin{array}{cc} H: & \;\theta \in \left\{\theta_{0}\right\}\times \Theta_{2}\\ A: & \;\theta \notin \left\{\theta_{0}\right\}\times \Theta_{2} \end{array}\;and\;\begin{array}{cc} \bar{H}: & \;\theta_{1}=\theta_{0}\\ \bar{A}: & \;\theta_{1}\neq \theta_{0}, \end{array}$$

respectively. Assume $\theta =\left(\theta_{1},\theta_{2}\right)$ is absolutely continuous with prior density function π, with $\theta_{1}⫫\theta_{2}$. Then, $\theta_{2}$ is a Non-Informative Nuisance Parameter for testing $\bar{H}$ versus $\bar{A}$ by using ${\bar{\varphi }}^{*}$, and for every x∈X such that (1) holds,

(23) $$\varphi^{*}\left(x\right)\;=\;1\;\;\Leftrightarrow \;\;{\bar{\varphi }}^{*}\left(x,\theta_{2}\right)\;=\;1$$

Theorem 3 tells us that when the likelihood function may be factored as (1), the mixed test obeys the NNPP. That is to say, if one aims to test a simple hypothesis about the parameter of interest $\theta_{1}$ in the presence of a non-informative nuisance parameter $\theta_{2}$ by means of the mixed test, then he can disregard $\theta_{2}$ in the analysis. Under a purely mathematical viewpoint, when x∈X satisfying (1) is observed, the decision between rejecting and accepting the null hypothesis regarding $\theta_{1}$ depends on $L_{x}$ only through the factor $L_{x}^{1}$, which is not a function of $\theta_{2}$, as we can see from Equation (A16) in Appendix A. It should be emphasized that Theorem 3 holds for null hypotheses more general than only simple ones. For instance, the Theorem is still valid when the null hypothesis *H* is of the form $H:\theta \in \Theta_{0}\times \Theta_{2}$, where $\Theta_{0}\subset \Theta_{1}$ is a hyperplane of $\Theta_{1}$. The proof of this result is quite similar to the proof of Theorem 3 in Appendix A and for this reason is omitted.

The adherence to the NNPP is indeed an advantage of the mixed test. It may bring a considerable reduction in the calculations involved along the procedure of the mixed test, especially under statistical models for which a statistic s-ancillary for the parameter of interest can be found. Such cases are examined after Corollary 1, which follows straightforwardly from Theorems 2 and 3.

**Corollary** **1.** Assume the same conditions of Theorem 3 and suppose that ∃T:X→T such that T is p-sufficient for $\theta_{2}$ and s-ancillary for $\theta_{1}$. Then, for all x∈X, $\varphi^{*}\left(x\right)=1\Leftrightarrow {\bar{\varphi }}^{*}\left(x,\theta_{2}\right)=1$.

Now, let us suppose that under experiment E=(X,θ,P), there is a statistic T:X→T such that *T* is s-ancillary for $\theta_{1}$. Let $H:\theta \in \left\{\theta_{0}\right\}\times \Theta_{2}$ be the hypothesis of interest. From the predictive distribution $f_{H}$ for *X*, we can define for each value t∈T(X)={T(x):x∈X} the conditional probability function for *X* given T(X)=t, $f_{H,t}$ by

(24) $$f_{H,t}\left(x\right)\;=\;\frac{f_{H}\left(x\right)}{\sum_{y\in X:T(y)=t}f_{H}\left(y\right)}\;$$

if T(x)=t, and $f_{H,t}\left(x\right)=0$, otherwise.

Finally, from the conditional distribution in (24), we define two conditional statistics: the conditional *p*-value and the conditional adaptive significance level. Such quantities will be of great importance for the performance of the mixed test, as we will see in the next section.

**Definition** **3.** ***Conditional p-value**: Let E=(X,θ,P) be an experiment for which the statistic T:X→T is s-ancillary for $\theta_{1}$. Let $H:\theta \in \left\{\theta_{0}\right\}\times \Theta_{2}$ be the hypothesis of interest, and $f_{H,t}$, t∈T(X), as in (24). We define the p-value conditional on T, $p_{T}-value:X\to \left[0,1\right]$ for each $x_{0}\in X$ by*

(25) $$p_{T}-value\left(x_{0}\right)\;=\;\sum_{x\;\in \;D(x_{0})}f_{H,T(x_{0})}\left(x\right)\;=\;\frac{\sum_{x\;\in \;D_{T}^{*}\left(x_{0}\right)}f_{H}\left(x\right)}{\sum_{x\;\in \;D_{T}\left(x_{0}\right)}f_{H}\left(x\right)}\;,$$

where $D_{T}^{*}\left(x_{0}\right)=\left\{x\in X:BF\left(x\right)\leq BF\left(x_{0}\right),T\left(x\right)=T\left(x_{0}\right)\right\}$ and $D_{T}\left(x_{0}\right)=\left\{x\in X:T\left(x\right)=T\left(x_{0}\right)\right\}$. From Equation (A14), the $P_{T}-value$ may be rewritten as

(26) $$p_{T}-value\left(x_{0}\right)\;=\;\frac{\sum_{x\;\in \;D_{T}^{*}\left(x_{0}\right)}\left[L_{x}^{1}\left(\theta_{0}\right)\int_{\Theta_{2}}L_{x}^{2}\left(\theta_{2}\right)\pi_{2}\left(\theta_{2}\right)d\theta_{2}\right]}{\sum_{x\;\in \;D_{T}\left(x_{0}\right)}\left[L_{x}^{1}\left(\theta_{0}\right)\int_{\Theta_{2}}L_{x}^{2}\left(\theta_{2}\right)\pi_{2}\left(\theta_{2}\right)d\theta_{2}\right]}\;,$$

*where $L_{x}^{1}\left(\theta_{0}\right)=P\left(X=x|\;T\left(X\right)=T\left(x\right),\theta_{0}\right)$ and $L_{x}^{2}\left(\theta_{2}\right)=P\left(T\left(X\right)=T\left(x\right)|\;\theta_{2}\right)$ since T is s-ancillary for $\theta_{1}$. It follows that*

$$\begin{array}{c} p_{T}-value\left(x_{0}\right)\;=\;\frac{\sum_{x\;\in \;D_{T}^{*}\left(x_{0}\right)}\left[P\left(X=x|\;T\left(X\right)=T\left(x\right),\theta_{0}\right)\int_{\Theta_{2}}P\left(T\left(X\right)=T\left(x\right)|\;\theta_{2}\right)\pi_{2}\left(\theta_{2}\right)d\theta_{2}\right]}{\sum_{x\;\in \;D_{T}\left(x_{0}\right)}\left[P\left(X=x|\;T\left(X\right)=T\left(x\right),\theta_{0}\right)\int_{\Theta_{2}}P\left(T\left(X\right)=T\left(x\right)|\;\theta_{2}\right)\pi_{2}\left(\theta_{2}\right)d\theta_{2}\right]}\\ \;\;\;\;\;\;\;\;=\;\frac{\sum_{x\;\in \;D_{T}^{*}\left(x_{0}\right)}\left[P\left(X=x|\;T\left(X\right)=T\left(x_{0}\right),\theta_{0}\right)\int_{\Theta_{2}}P\left(T\left(X\right)=T\left(x_{0}\right)|\;\theta_{2}\right)\pi_{2}\left(\theta_{2}\right)d\theta_{2}\right]}{\sum_{x\;\in \;D_{T}\left(x_{0}\right)}\left[P\left(X=x|\;T\left(X\right)=T\left(x_{0}\right),\theta_{0}\right)\int_{\Theta_{2}}P\left(T\left(X\right)=T\left(x_{0}\right)|\;\theta_{2}\right)\pi_{2}\left(\theta_{2}\right)d\theta_{2}\right]}\;=\\ \;\;=\;\frac{\sum_{x\;\in \;D_{T}^{*}\left(x_{0}\right)}P\left(X=x|\;T\left(X\right)=T\left(x_{0}\right),\theta_{0}\right)}{\sum_{x\;\in \;D_{T}\left(x_{0}\right)}P\left(X=x|\;T\left(X\right)=T\left(x_{0}\right),\theta_{0}\right)}; \end{array}$$

*that is*,

(27) $$p_{T}-value\left(x_{0}\right)\;=\;\sum_{x\;\in \;D_{T}^{*}\left(x_{0}\right)}P\left(X=x|\;T\left(X\right)=T\left(x_{0}\right),\theta_{0}\right)\;.$$

**Definition** **4.** ***Conditional adaptive significance level*** : Let E=(X,θ,P) be an experiment for which the statistic T:X→T is s-ancillary for $\theta_{1}$. Let $H:\theta \in \left\{\theta_{0}\right\}\times \Theta_{2}$ be the hypothesis of interest and $f_{H,t}$, t∈T(X) be as in (24). We define the conditional adaptive significance level given T, $\alpha_{T}^{*}:X\to \left[0,1\right]$, for each $x_{0}\in X$ by

(28) $$\alpha_{T}^{*}\left(x_{0}\right)\;=\;\sum_{x\;\in \;D}f_{H,T(x_{0})}\left(x\right)\;=\;\frac{\sum_{x\;\in \;D\cap D_{T}\left(x_{0}\right)}f_{H}\left(x\right)}{\sum_{x\;\in \;D_{T}\left(x_{0}\right)}f_{H}\left(x\right)}\;.$$

The conditional adaptive significance level $\alpha_{T}^{*}$ may be rewritten as

(29) $$\alpha_{T}^{*}\left(x_{0}\right)\;=\;\sum_{x\;\in \;D\cap D_{T}\left(x_{0}\right)}P\left(X=x|\;T\left(X\right)=T\left(x_{0}\right),\theta_{0}\right)\;.$$

Definitions 3 and 4 are conditional versions of Definitions in (18) and (19), respectively. While calculation of the unconditional quantities involves the evaluation of the Bayes factor for every x∈X, the determination of the conditional statistics at a specific sample point $x_{0}\in X$ depends only on the values of the Bayes factor for the sample points *x* such that $T\left(x\right)=T\left(x_{0}\right)$, which may be much easier to accomplish. Note also that the $p_{T}$-value and $\alpha_{T}^{*}$ can be seen, respectively, as an alternative (conditional) measure of evidence in favor of the null hypothesis *H* and an alternative threshold value for testing the competing hypotheses. As a matter of fact, one can substitute the *p*-value and the adaptive significance level with their conditional versions in order to perform the mixed test. This is exactly what the next theorem states.

**Theorem** **4.** *Assume the same conditions as in Corollary 1 and Theorem 3. Then, for all $x_{0}\in X$*,

$$\varphi^{*}\left(x_{0}\right)\;=\;1\;\;\Leftrightarrow \;\;p_{T}-value\left(x_{0}\right)⩽\alpha_{T}^{*}\left(x_{0}\right)\;.$$

The results of Theorems 3 and 4 and Corollary 1 suggest a way the mixed test may be used without doing so many calculations: when an ancillary statistic for the parameter of interest, *T*, is available, one can perform the test by comparing the conditional statistics $p_{T}-value$ and $\alpha_{T}^{*}$ instead of comparing the unconditional ones in Definitions (18) and (19). This possibility is illustrated in the next section.

## 4. Examples

We now revisit three well-known problems of hypothesis testing for count data and present new solutions to them by means of the mixed test. In each problem, we consider a suitable reparametrization of the standard model in order to ensure that

1. There exists a statistic *T* that is ancillary to the new parameter of interest;
2. The hypothesis about the new parameter of interest under the reparametrization is a singleton (or a hyperplane);
3. The new parameter of interest is independent of the new nuisance parameter a priori;
4. The distribution of the data *X* given any value of the statistic *T* is simple enough to render the calculations of the conditional *p*-value and the conditional adaptive significance level easy.

### 4.1. Comparison of Poisson Means

Suppose we are interested in testing the equality between two Poisson means: say $\theta_{1}$ and $\theta_{2}$. Let $\theta =(\theta_{1},\theta_{2})$. For this purpose, let $X=(X_{1},X_{2})$ be a random vector to be observed such that given θ, $X_{1}$ and $X_{2}$ are independent Poisson random variables with parameters $n\theta_{1}$ and $n\theta_{2}$, respectively, where $n\in N^{*}=\left\{1,2,3,\ldots \right\}$ is a known integer. The hypotheses to be tested are

(30) $$\begin{array}{cc} H: & \;\theta \in \Theta_{0}\\ A: & \;\theta \in \Theta_{0}^{c}, \end{array}$$

where $\Theta_{0}=\left\{\left(\theta_{1},\theta_{2}\right)\in R_{+}^{2}:\theta_{1}=\theta_{2}\right\}$. The likelihood function for $\theta \in R_{+}^{2}$ generated by $x=\left(x_{1},x_{2}\right)\in N^{2}$ is

(31) $$L_{x}\left(\theta \right)=\frac{{\left(n\theta_{1}\right)}^{x_{1}}}{x_{1}!}\frac{{\left(n\theta_{2}\right)}^{x_{2}}}{x_{2}!}e^{-\theta_{1}n}e^{-\theta_{2}n}.$$

Suppose also that $\theta_{1}$ and $\theta_{2}$ are independent a priori and that $\theta_{i}$ is distributed as a Gamma random variable with parameters $a_{i}>0$ and c>0, i=1,2. That is, the prior density function of θ is

(32) $$\pi \left(\theta \right)=\frac{c^{a_{1}}}{\Gamma (a_{1})}\theta_{1}^{a_{1}-1}e^{-c\theta_{1}}I_{\left(0,\infty \right)}\left(\theta_{1}\right)\frac{c^{a_{2}}}{\Gamma (a_{2})}\theta_{2}^{a_{2}-1}e^{-c\theta_{2}}I_{\left(0,\infty \right)}\left(\theta_{2}\right).$$

Although one can determine an exact expression for the Bayes factor in this case (as a matter of fact, in [25], the authors first presented a solution to the problem of testing the equality of Poisson means by using weighted likelihoods in the context of a production process monitoring procedure), the use of the mixed test under the above parametrization may be computationally disadvantageous, as the sample space is $N^{2}$ and one should determine infinitely many Bayes factors to perform the test. To overcome this difficulty, we next consider the following reparametrization of the model: let $\lambda =(\lambda_{1},\lambda_{2})$ be the new parameter, where

(33) $$\lambda_{1}=\frac{\theta_{1}}{\theta_{1}+\theta_{2}}\;and\;\lambda_{2}=\theta_{1}+\theta_{2}.$$

The new parameter space is then $\Lambda =\left(0,1\right)\times R_{+}$. Now, the hypotheses (30) can be rewritten as

(34) $$\begin{array}{cc} \tilde{H}: & \;\lambda \in \Lambda_{0}\\ \tilde{A}: & \;\lambda \in \Lambda_{0}^{c}, \end{array}$$

with $\Lambda_{0}=\left\{\frac{1}{2}\right\}\times R_{+}$. Note that the likelihood function (31) can be rewritten by conditioning on the statistic $T\left(X\right)=X_{1}+X_{2}$ as follows:

(35) Lx(θ)=P(X1=x1,X2=x2|θ)=P(X1=x1,X2=x2|X1+X2=x1+x2,θ)P(X1+X2=x1+x2|θ)=x1+x2x1θ1θ1+θ2x1θ2θ1+θ2x2e−n(θ1+θ2)[n(θ1+θ2)]x1+x2(x1+x2)!.

Hence, the induced likelihood function for λ generated by $\left(x_{1},x_{2}\right)$ may be factored as

(36) L˜x(λ)=x1+x2x1λ1x11−λ1x2(nλ2)x1+x2(x1+x2)!e−λ2n

Note that *T* is an ancillary statistic for $\lambda_{1}$, as it is distributed as a Poisson random variable with mean $n\lambda_{2}$, and the conditional distribution of *X*, given T(X)=t, t∈N, depends on λ only through $\lambda_{1}$. The prior distribution for λ is given by

(37) $$\tilde{\pi }\left(\lambda \right)=\frac{\Gamma (a_{1}+a_{2})}{\Gamma \left(a_{1}\right)\Gamma \left(a_{2}\right)}\lambda_{1}^{a_{1}-1}{\left(1-\lambda_{1}\right)}^{a_{2}-1}I_{\left(0,1\right)}\left(\lambda_{1}\right)\frac{c^{a_{1}+a_{2}}}{\Gamma (a_{1}+a_{2})}\lambda_{2}^{a_{1}+a_{2}-1}e^{-c\lambda_{2}}I_{\left(0,\infty \right)}\left(\lambda_{2}\right)\;.$$

Now, as (34), (36) and (37) hold, it follows from Theorem 3 that $\lambda_{2}$ is an NNP and that the performance of the mixed test for the hypothesis $\tilde{H}:\lambda \in \left\{\frac{1}{2}\right\}\times R_{+}$ against $\tilde{A}$ based on ${\tilde{L}}_{x}$ and the prior $\tilde{\pi }$ is equivalent to the performance of the mixed test for the simple hypothesis $\lambda_{1}=\frac{1}{2}$ against $\lambda_{1}\neq \frac{1}{2}$ based on the binomial-like factor of ${\tilde{L}}_{x}$ that depends only on $\theta_{1}$ and the marginal Beta prior density for $\lambda_{1}$ ignoring the NNP $\lambda_{2}$. In addition, Theorem 4 implies that the test for $\tilde{H}$ versus $\tilde{A}$ reduces to the comparison of the statistics $p_{T}$-value and $\alpha_{T}^{*}$ at the observed sample point, say $x_{0}=\left(x_{01},x_{02}\right)$. Note that in this case, one does not need to evaluate the Bayes factor for every point of $N^{2}$ but only for those $x_{01}+x_{02}+1$ of them for which the sum of the components is $x_{01}+x_{02}$. That is, one needs to evaluate the Bayes factor only for the elements of $\left\{\left(u,v\right)\in N^{2}:T\left(u,v\right)=T\left(x_{0}\right)=x_{01}+x_{02}\right\}$ when $x_{0}=\left(x_{01},x_{02}\right)$ is observed.

From Equations (A14) and (A15), one gets the following predictive functions under $\tilde{H}$ and under $\tilde{A}$ for *X*:

(38) fH˜(x)=(1/2)x1+x2x1+x2x1×K

and

(39) fA˜(x)=x1+x2x1Γ(x1+a1)Γ(x2+a2)Γ(x1+x2+a1+a2)Γ(a1+a2)Γ(a1)Γ(a2)×K,

where $K=\int_{0}^{\infty }\left[\frac{{\left(n\lambda_{2}\right)}^{x_{1}+x_{2}}}{(x_{1}+x_{2})!}e^{-\lambda_{2}n}\right]\frac{c^{a_{1}+a_{2}}}{\Gamma (a_{1}+a_{2})}\lambda_{2}^{a_{1}+a_{2}-1}e^{-c\lambda_{2}}d\lambda_{2}$. Consequently, the Bayes factor BF(x) is

(40) $$\begin{array}{cc} BF(x)= & {\left(1/2\right)}^{x_{1}+x_{2}}\frac{\Gamma \left(a_{1}\right)\Gamma \left(a_{2}\right)}{\Gamma (a_{1}+a_{2})}\frac{\Gamma (x_{1}+x_{2}+a_{1}+a_{2})}{\Gamma \left(x_{1}+a_{1}\right)\Gamma \left(x_{2}+a_{2}\right)}. \end{array}$$

Finally, it follows from (28) and (30) that for $x_{0}=\left(x_{01},x_{02}\right)\in N^{2}$,

(41) pT-valuex0=∑(x1,x2)∈DT*(x0)x1+x2x1(1/2)x1+x2=∑x1=0T(x0)T(x0)x1(1/2)T(x0)ID(x0)(x1,T(x0)−x1)

and

(42) αT*x0=∑(x1,x2)∈D∩DT(x0)x1+x2x1(1/2)x1+x2=∑x1=0T(x0)T(x0)x1(1/2)T(x0)ID(x1,T(x0)−x1).

Note that in this case, the conditional $p_{T}$-value resembles the frequentist *p*-value for the simple hypothesis $\theta =\frac{1}{2}$ under simple random sampling from the Bernoulli model with parameter θ (however, for the calculation of the $p_{T}$-value, the sample space is ordered by the Bayes factor instead of the likelihood ratio).

**Example** **3** (**Comparison of Poisson means**). *In [25], the authors consider that a methodology to detect a shift in a production process is to compare the quality index of the current rating period P, $\theta_{2}$, with the quality index of the previous rating period, $\theta_{1}$. Suppose that we want to test if a process is under control; that is, if $\theta_{1}=\theta_{2}$. For this purpose, two audit samples of size n=10 are collected at rating periods P−1 and P, respectively. Let $X_{1}$ represent the number of defects found in the first sample and $X_{2}$ represent the number of defects found in the second sample. Also suppose that $X_{1}$ and $X_{2}$ are Poisson random variables with parameters $n\theta_{1}$ and $n\theta_{2}$, respectively. Let $X=(X_{1},X_{2})$. For simplicity, we consider the hyperparameters in (32) as $a_{1}=a_{2}=c=1$. Hence, the predictive functions under the competing hypothesis are given by:*

(43) fH(x)=(1/2)x1+x2(x1+x2+1)x1+x2x1nn+1x1+x21n+12,

*and*

(44) $$f_{A}\left(x\right)={\left(\frac{n}{n+1}\right)}^{x_{1}+x_{2}}{\left(\frac{1}{n+1}\right)}^{2}.$$

*Consequently, the Bayes factor at $x=\left(x_{1},x_{2}\right)\in N^{2}$ can be expressed by*

(45) BF(x)=(1/2)x1+x2(x1+x2+1)x1+x2x1.

*Now, suppose that two defects are found at rating period P−1 and nine defects are found at period P. That is, suppose that X=(2,9) is observed. In this case, one gets BF(2,9)=0.322. Considering $\frac{b}{a}=1$, the conditional adaptive significance level and the conditional $p_{T}$-value at (2,9) are, respectively*,

αT*((2,9))=∑x1∈{0,1,2,3,8,9,10,11}2+9x1(1/2)2+9=0.227and

PT-value((2,9))=∑x1∈{0,1,2,9,10,11}11x1(1/2)11=0.065.

Since $P_{T}-\mathrm{value}\left(\left(2,9\right)\right)<\alpha_{T}^{*}\left(\left(2,9\right)\right)$, the decision is to reject the null hypothesis (34), where $\lambda =(\frac{\theta_{1}}{\theta_{1}+\theta_{2}},\theta_{1}+\theta_{2})$.

Note that although the sample size is small, the null hypothesis can be rejected with a conditional $P_{T}-value$ of 0.065. Such a value is not compared with standard (fixed) cut-off values such as 0.01 or 0.05 but rather with the conditional adaptive significance level of 0.227 for X=(2,9). Note also that performance of the mixed test by means of the conditional statistics $p_{T}$-value and $\alpha_{T}^{*}$ when X=(2,9) is observed requires the calculation of only finitely many Bayes factors (twelve, precisely) even though the sample space is infinite.

### 4.2. Test of Symmetry

Suppose we want to test the hypothesis of symmetry in an r×r two-way contingency table. Several methods have been proposed for testing diagonal symmetry: see, for example, [28,29,30,31,32,33] and references therein. Here we propose a solution to this problem by using the mixed test and its properties. We present the simplest case r=2. The reader will find the general case r>2 in Appendix A for the sake of readability.

Suppose each element (individual) of a sample of size *n* is classified into four mutually exclusive combinations of the two-valued variables $X_{1}$ and $X_{2}$. Let Table 1 represent the observed frequencies of the cross-classifications, where $X_{ij}$ is the number of individuals classified into the i-th category of $X_{1}$ and the j-th category of $X_{2}$, i,j=1,2. Let $\theta =(\theta_{11},$$\theta_{12},\theta_{21})$, with $\theta_{ij}\geq 0$ and $\theta_{11}+\theta_{12}+\theta_{21}\leq 1$, where $\theta_{ij}$ denotes the probability of classification into the i-th category of $X_{1}$ and the j-th category of $X_{2}$, i,j=1,2.

The hypotheses for testing diagonal symmetry are

(46) $$\begin{array}{cc} H: & \theta_{12}=\theta_{21}\\ A: & \theta_{12}\neq \theta_{21} \end{array}$$

We assume that the vector $X=(X_{11},X_{12},X_{21})$ is, given θ, a multinomial random vector with parameters *n* and θ. The likelihood function generated by $x=(x_{11},x_{12},x_{21})$ is then given by

(47) $$L_{x}\left(\theta \right)\;=\;\frac{n!}{x_{11}!x_{12}!x_{21}!x_{22}!}\theta_{11}^{x_{11}}\theta_{12}^{x_{12}}\theta_{21}^{x_{21}}\theta_{22}^{x_{22}}\;,$$

where $x_{22}=n-x_{11}-x_{12}-x_{21}$ and $\theta_{22}=1-\theta_{11}-\theta_{12}-\theta_{21}$. Assume also a prior Dirichlet distribution with parameter vector $\alpha =(\alpha_{11},\alpha_{12},\alpha_{21};\alpha_{22})$, $\alpha_{ij}>0$ for θ. That is,

(48) $$\pi \left(\theta \right)\;=\;\frac{\Gamma (\alpha_{11}+\alpha_{12}+\alpha_{21}+\alpha_{22})}{\Gamma \left(\alpha_{11}\right)\Gamma \left(\alpha_{12}\right)\Gamma \left(\alpha_{21}\right)\Gamma \left(\alpha_{22}\right)}\theta_{11}^{\alpha_{11}-1}\theta_{12}^{\alpha_{12}-1}\theta_{21}^{\alpha_{21}-1}\theta_{22}^{\alpha_{22}-1}I_{\Theta }\left(\theta \right)\;,$$

where $\Theta =\{\left(u_{1},u_{2},u_{3}\right)\in R_{+}^{3}:u_{1}+u_{2}+u_{3}\leq 1\}$.

We should note that the determination of the predictive functions is much easier under the following reparametrization of the model: let us define

$$\lambda_{11}\;=\;\theta_{11}\;\;\;\lambda_{21}\;=\;\theta_{12}+\theta_{21}\;\;\;\mathrm{and}\;\;\;\lambda_{12}\;=\;\frac{\theta_{12}}{\theta_{12}+\theta_{21}}\;.$$

Let $\lambda =(\lambda_{12},\left(\lambda_{11},\lambda_{21}\right))$. Thus, the new parameter space is $\Lambda =\left(0,1\right)\times S_{2}$ , where $S_{2}=\left\{\left(u,v\right)\in R_{+}^{2}:u+v\leq 1\right\}$.

Then, we can reformulate the hypotheses (46) as

(49) $$\begin{array}{cc} \tilde{H}: & \lambda \in \Lambda_{0}\\ \tilde{A}: & \lambda \in \Lambda_{0}^{c}\;, \end{array}$$

where $\Lambda_{0}=\left\{\frac{1}{2}\right\}\times S_{2}$. Note that the likelihood function for θ generated by $x=(x_{11},$$x_{12},x_{21})\in \left\{\left(a,b,c\right)\in N^{3}:a+b+c\leq n\right\}$ can be rewritten by conditioning on the statistic $T\left(X\right)=\left(X_{11},X_{12}+X_{21}\right)$ as

(50) Lx(θ)=P(X=(x11,x12,x21)|T(X)=(x11,x12+x21),θ)P(T(X)=(x11,x12+x21)|θ)=x12+x21x12θ12θ12+θ21x12θ21θ12+θ21x21n!x11!(x12+x21)!x22!θ11x11(θ12+θ21)x12+x21θ22x22.

Hence, the induced likelihood function for λ generated by $x=(x_{11},x_{12},x_{21})$ may be factored as

(51) L˜x(λ)=x12+x21x12λ12x121−λ12x21n!x11!(x12+x21)!x22!λ11x11λ21x12+x21(1−λ11−λ21)x22

Note that *T* is an ancillary statistic for $\lambda_{12}$ as it is a multinomial random vector with parameters *n* and $\left(\lambda_{11},\lambda_{21}\right)$, and the conditional distribution of *X* given T(X)=t, $t\in \{\left(u,v\right)\in N^{2}:u+v\leq n\}$ depends on λ only through $\lambda_{12}$. The prior distribution for λ is given by

(52) $$\tilde{\pi }\left(\lambda \right)=\frac{\Gamma (\alpha_{12}+\alpha_{21})}{\Gamma \left(\alpha_{12}\right)\Gamma \left(\alpha_{21}\right)}\lambda_{12}^{\alpha_{12}-1}{\left(1-\lambda_{12}\right)}^{\alpha_{21}-1}I_{\left(0,1\right)}\left(\lambda_{12}\right)\;{\tilde{\pi }}_{\left(\lambda_{11},\lambda_{21}\right)}\left(\lambda_{11},\lambda_{21}\right)\;,$$

where ${\tilde{\pi }}_{\left(\lambda_{11},\lambda_{21}\right)}$ is the prior Dirichlet distribution for $\left(\lambda_{11},\lambda_{21}\right)$ with parameter vector $\left(\alpha_{11},\alpha_{12}+\alpha_{21},\alpha_{22}\right)$.

As in the example of the previous subsection, the results from Section 3 imply that $\left(\lambda_{11},\lambda_{21}\right)$ is an NNP for testing the hypotheses $\tilde{H}$ versus $\tilde{A}$ in (50) by using the mixed test. In addition, we only need to compare the conditional $p_{T}$-value with the conditional adaptive significance level to decide between the hypotheses.

From Equations (A14) and (A15), one gets the following predictive functions under $\tilde{H}$ and under $\tilde{A}$ for *X*:

(53) fH˜(x11,x12,x21)=(1/2)x12+x21x12+x21x12×K

and

(54) fA˜(x)=x12+x21x12Γ(x12+α12)Γ(x21+α21)Γ(x12+x21+α12+α21)Γ(α12+α21)Γ(α12)Γ(α21)×K,

where

$$K=\int_{S_{2}}\left[\frac{n!}{x_{11}!\left(x_{12}+x_{21}\right)!x_{22}!}\lambda_{11}^{x_{11}}\lambda_{21}^{x_{12}+x_{21}}{\left(1-\lambda_{11}-\lambda_{21}\right)}^{x_{22}}\right]{\tilde{\pi }}_{\left(\lambda_{11},\lambda_{21}\right)}\left(\lambda_{11},\lambda_{21}\right)d\lambda_{11}d\lambda_{21}.$$

Consequently, the Bayes factor BF(x) is

(55) $$\begin{array}{cc} BF(x)\;= & \;{\left(1/2\right)}^{x_{12}+x_{21}}\frac{\Gamma \left(\alpha_{12}\right)\Gamma \left(\alpha_{21}\right)}{\Gamma (\alpha_{12}+\alpha_{21})}\frac{\Gamma (x_{12}+x_{21}+\alpha_{12}+\alpha_{21})}{\Gamma \left(x_{12}+\alpha_{12}\right)\Gamma \left(x_{21}+\alpha_{21}\right)}. \end{array}$$

Finally, it follows from (28) and (30) that for $x=(x_{11},x_{12},x_{21})$,

(56) pT-valuex=∑(y11,y12,y21)∈DT*(x)y12+y21y12(1/2)y12+y21=∑y12=0x12+x21x12+x21y12(1/2)x12+x21ID(x)(x11,y12,x12+x21−y12)

and

(57) αT*x=∑(y11,y12,y21)∈D∩DT(x)y12+y21y12(1/2)y12+y21=∑y12=0x12+x21x12+x21y12(1/2)x12+x21ID(x11,y12,x12+x21−y12)

In this example (as in the previous subsection), the conditional $p_{T}$-value looks like the frequentist *p*-value for the simple hypothesis $\theta =\frac{1}{2}$ regarding an unknown proportion. We should emphasize that for calculation of the $p_{T}$-value, the sample space is ordered by the Bayes factor in place of the likelihood ratio. Note also that the evaluation of this conditional statistic involves ordering at most n+1 points of the sample space (exactly those for which the statistic *T* takes the value $T(x_{0})$ when $x_{0}$ is the effectively observed sample point). On the other hand, if one performs the mixed test without using these conditional quantities, he shall order all n+33=(n+3)(n+2)(n+1)6 elements of the sample space.

**Example** **4** (**Analysis of opinion swing**). *Suppose it is of interest to evaluate whether the proportion of individuals that did not support the US President before the State of the Union Address remained unchanged after his address. For this purpose, n=100 individuals are surveyed with regard to their support for the President before and after his annual message. The survey results are displayed in the following 2×2 contingency Table 2:*

*Let $X_{1}$ ($X_{2}$) be the support—“No” or “Yes”—for the President before (after) the State of the Union Address. Let $\theta_{ij}$ be the probability that an individual is classified into the i-th category of $X_{1}$ and j-th category of $X_{2}$ (for instance, $\theta_{11}$ is the probability that an individual does not support the President both before and after his address). The hypothesis that the support for the President remains unchanged is $\theta_{21}+\theta_{22}=\theta_{12}+\theta_{22}$. This is equivalent to the hypothesis that the proportion of swings from “Yes” to “No” is equal to the proportion of swings from “No” to “Yes”; that is, this is equivalent to the symmetry hypothesis $\theta_{12}=\theta_{21}$. Thus, we can test such a hypothesis by means of the mixed test considering the mathematical setup of this subsection. Suppose α=(1,1,1,1). Then, the Bayes factor is given by*

(58) BF(x11,x12,x21)=(1/2)x12+x21(x12+x21+1)x12+x21x12.

*For the observed data x=(20,17,10), we obtain the Bayes factor BF((20,17,10))=1.76. Considering a=b (that is b/a=1), we do not reject the null hypothesis since BF((20,17,10))>1. In this case, the conditional adaptive significance level and the conditional $P_{T}-\mathrm{value}$ at the point $\left((20,17,10)\right)$ are*

αT*((20,17,10))=∑y12∈{0,…,9}∪{18,…,27}17+10y12(1/2)17+10=0.122and

PT-value((20,17,10))=∑y12∈{0,…,10}∪{17,…,27}27y12(1/2)27=0.248.

Note that $p_{T}$-value$\left(\left(20,17,10\right)\right)\;>\;\alpha_{T}^{*}\left(\left(20,17,10\right)\right)$, as it was expected. Note also that we ordered only 28 elements of the sample space by the Bayes factor to determine the above conditional quantities. To calculate the unconditional ones, we should have ordered all 176,851 points in the sample space.

### 4.3. Test of Independence

Consider the same statistical model as in the previous subsection. However, now we want to evaluate whether there exists (or not) association between the variables $X_{1}$ and $X_{2}$. For this purpose, we may test the independence hypothesis between these variables. Consider the joint distribution for $\left(X_{1},X_{2}\right)$ in Table 3 below:

The hypotheses to be tested are

(59) $$\begin{array}{cc} H: & \;\theta_{11}\;=\;\left(\theta_{11}+\theta_{12}\right)\left(\theta_{11}+\theta_{21}\right)\\ A: & \;\theta_{11}\;\neq \;\left(\theta_{11}+\theta_{12}\right)\left(\theta_{11}+\theta_{21}\right) \end{array}$$

It is easy to check that hypotheses *H* and *A* can be rewritten as

(60) $$\begin{array}{cc} H: & \;\frac{\theta_{11}}{\theta_{11}+\theta_{12}}\;=\;\frac{\theta_{21}}{1-(\theta_{11}+\theta_{12})}\\ A: & \;\frac{\theta_{11}}{\theta_{11}+\theta_{12}}\;\neq \;\frac{\theta_{21}}{1-(\theta_{11}+\theta_{12})} \end{array}$$

Let us define

$$\lambda_{11}\;=\;\frac{\theta_{11}}{\theta_{11}+\theta_{12}}\;\;\lambda_{21}\;=\;\frac{\theta_{21}}{1-(\theta_{11}+\theta_{12})}\;\;\mathrm{and}\;\;\lambda_{12}\;=\;\theta_{11}+\theta_{12}\;$$

and consider the new parameter $\lambda =(\left(\lambda_{11},\lambda_{21}\right),\lambda_{12})$, which takes value in $\Lambda ={\left(0,1\right)}^{2}\times \left(0,1\right)$. Let $T\left(X\right)=T\left(X_{11},X_{12},X_{21}\right)=X_{11}+X_{12}$. Proceeding as in the previous subsections, we obtain the following induced likelihood function for λ generated by $x=(X_{11},x_{12},x_{21})$

(61) L˜x(λ)=x11+x12x11λ11x111−λ11x12x21+x22x21λ21x211−λ21x22nx11+x12λ12x11+x121−λ12x21+x22

Note that *T* is an ancillary statistic for $\left(\lambda_{11},\lambda_{21}\right)$, as it is a binomial random variable with parameters *n* and $\lambda_{12}$. In addition, for each possible value *t* of the statistic *T*, the conditional distribution of *X* given T(X)=t depends on λ only through $\left(\lambda_{11},\lambda_{21}\right)$.

The prior distribution for λ is such that $\lambda_{11}$, $\lambda_{21}$ and $\lambda_{12}$ are independent Beta random variables with respective parameters $\alpha_{11}$ and $\alpha_{12}$, $\alpha_{21}$ and $\alpha_{22}$, and $\alpha_{11}+\alpha_{12}$ and $\alpha_{21}+\alpha_{22}$.

Finally, note that under the new parametrization, the independence hypothesis is

(62) $$\begin{array}{cc} \tilde{H}: & \lambda \in \Lambda_{0}\\ \tilde{A}: & \lambda \notin \Lambda_{0}\;, \end{array}$$

where $\Lambda_{0}=\left\{\left(u,v\right)\in {\left(0,1\right)}^{2}:u=v\right\}\times \left(0,1\right)$.

From the results from Section 3, it follows that $\lambda_{12}$ is an NNP for testing the hypotheses $\tilde{H}$ versus $\tilde{A}$ above by using the mixed test. In addition, we only need to compare the conditional $P_{T}-value$ with the conditional adaptive significance level to decide between these hypotheses. In a sense, the test for the hypothesis of independence between $X_{1}$ and $X_{2}$ by using the conditional statistics resembles the test for the hypothesis of homogeneity were the marginal counts $X_{11}+X_{12}$ and $n-X_{11}-X_{12}$ fixed beforehand.

Considering as in the previous section α=(1,1,1,1), we obtain the following expression for the Bayes factor:

BF(x11,x12,x21)=36(x11+x21+1)!(n+1−x11−x21)!x11!x12!x21!x22!(n+3)n+2x11+x21+1.

The conditional predictive probability function for *X* given $T\left(X\right)=X_{11}+X_{12}=t$, t=0,…,n is given by

(63) fH,t(x11,x12,x21)=6tx11n−tx21(n+3)n+2x11+x21+1IT−1(t)(x11,x12,x21),

where $T^{-1}\left(t\right)=\left\{\left(y_{11},y_{12},y_{21}\right)\in X:T\left(y_{11},y_{12},y_{21}\right)=y_{11}+y_{12}=t\right\}$.

From the above distribution, one may obtain the conditional $P_{T}-value$ and the conditional adaptive significance level $\alpha_{T}^{*}$ at each point in the sample space.

**Example** **5** (**Market’s directional change**). *In [34] it is argued that the directional change of the stock market in January signals the directional change of the market for the remainder of the year. Suppose the following Table 4 summarizes the directional changes of the prices of a few stocks in both periods*.

*In this case, the Bayes factor is given by*

(64) BF(x11,x12,x21)=36(x11+x21+1)!(16−x11−x21)!x11!x12!x21!x22!1817x11+x21+1.

*For the observed data x=(5,0,2), we obtain the Bayes factor BF((5,0,2))=0.244. Considering a=b (that is b/a=1), we reject the null hypothesis by using the mixed test since BF((5,0,2))<1. That is, the data from only a few stocks reveal that the directional change for the remainder of the year depends on the directional change in January. Note that although the sample size is small (n=15) and a cell count is equal to zero, the mixed test can be fully performed, as opposed to standard tests for the hypothesis of independence that rely on asymptotic results. In this case, the conditional adaptive significance level and the conditional $p_{T}$-value at the point $\left((5,0,2)\right)$ are*

$$\alpha_{T}^{*}\left(\left(5,0,2\right)\right)\;=\;0.0109\;\;\;and\;\;\;P_{T}-\mathrm{value}\left(\left(5,0,2\right)\right)\;=\;0.0022\;.$$

## 5. Discussion

Statistical hypothesis testing is an important quantitative method that may help the daily activity of scientists from different areas of knowledge. However, with recent computational advances, the misuse of standard tests have come to light. Thus, problems with tests of significance and fixed-level tests have brought a growing need for alternative approaches to hypothesis testing that do not have such drawbacks. Among these alternatives, we revisit in this manuscript the mixed test by Pericchi and Pereira, which combines aspects from two opposing viewpoints: the frequentist and the Bayesian. The mixed test satisfies various reasonable properties one desires when performing a test of hypotheses. Here we prove that the mixed test also meets the Non-Informative Nuisance Parameter Principle (NNPP) for simple hypotheses regarding the parameter of interest. The NNPP concerns the question of how to make inferences about a parameter in the presence of Non-Informative Nuisance Parameters: it states that it is irrelevant whether a Non-Informative Nuisance Parameter is known or unknown in order to draw conclusions about a quantity of interest from data. This principle, though important, has not been explored in some depth, and for this reason, we studied it further in hypothesis testing problems. Nuisance parameters typically affect inferences about a parameter of interest: when the variance is unknown, estimation of the mean of a normal distribution and estimation of the parameters of a linear regression model are examples of this.

Adherence of the mixed test to the NNPP allowed for much easier performance of the test, as the calculations involved were significantly reduced. Indeed, decision making between the competing statistical hypotheses was simplified in the three examples we examined: in each situation, conditioning on a suitable statistic and considering conditional versions of the *p*-value and the adaptive significance level were revealed to be an advantageous course of action to use the mixed test. The extent to which the adherence of the mixed test to the NNPP is valid and the use of the mixed test can then be made easier remains unanswered in this work. This issue is the goal of future investigation. 

## Figures and Tables

**Table 1:** Observed frequencies of $\left(X_{1},X_{2}\right)$ in the 2×2 case.

	$X_{2}$		
	$x_{11}$	$x_{12}$	$n_{1.}$
$X_{1}$	$x_{21}$	$x_{22}$	$n_{2.}$
	$n_{.1}$	$n_{.2}$	*n*

**Table 2:** Survey results.

		After		
		No	Yes	
	No	20	17	37
Before	Yes	10	53	63
		30	70	100

**Table 3:** Joint distribution of $X_{1}$ and $X_{2}$ given θ.

	$X_{2}$		
	$\theta_{11}$	$\theta_{12}$	$\theta_{1.}$
$X_{1}$	$\theta_{21}$	$\theta_{22}$	$\theta_{2.}$
	$\theta_{.1}$	$\theta_{.2}$	1

**Table 4:** Survey results.

		After January		
		Up	Down	
	up	5	0	5
January change	down	2	8	10
		7	8	15

## Data Availability

No new data were created or analyzed in this study. Data sharing is not applicable to this article.

## Appendix A

**Proof of Theorem** **2.** Suppose there exists a statistic T:X→T such that it is p-sufficient for $\theta_{1}$ and s-ancillary for $\theta_{2}$. Then,

(A1) $$\begin{array}{cc} P(X=x|\;\theta )= & P(X=x,T(X)=T(x)\;|\;\theta )\\ = & P\left(X=x|T\left(X\right)=T\left(x\right),\;\theta_{1},\theta_{2}\right)P\left(T\left(X\right)=T\left(x\right)\;|\;\theta_{1},\theta_{2}\right) \end{array}$$

The result is immediate: as the conditional distribution of *X* given T(X)=T(x) and θ depends only on $\theta_{2}$, and the marginal distribution of *T* given θ depends only on $\theta_{1}$, one can write for each x∈X, $P\left(T\left(X\right)=T\left(x\right)\;|\;\theta_{1},\theta_{2}\right)=L_{x}^{1}\left(\theta_{1}\right)$ and $P(X=x|T\left(X\right)=T\left(x\right)\;|\;\theta_{1},\theta_{2})=L_{x}^{2}\left(\theta_{2}\right)$. The proof when *T* is p-sufficient for $\theta_{2}$ and s-ancillary for $\theta_{1}$ is analogous. □

**Proof of Theorem** **1.** We first prove the (⇒) part of the theorem. Suppose that x∈X is such that $\theta_{1}⫫\theta_{2}\;|\;X=x$; that is, the posterior distribution of θ given X=x can be factored as $\pi \left(\theta |\;x\right)=\pi_{1}\left(\theta_{1}|\;x\right)\pi_{2}\left(\theta_{2}|\;x\right)$. Then,

(A2) $$\begin{array}{c} \;\pi \left(\theta |x\right)\;=\;\;\pi_{1}\left(\theta_{1}|x\right)\pi_{2}\left(\theta_{2}|x\right)\;\Rightarrow \;\frac{L_{x}\left(\theta \right)\pi \left(\theta \right)}{\int_{\Theta }L_{x}\left(\theta \right)\pi \left(\theta \right)d\theta }\;=\;\frac{\int_{\Theta_{2}}L_{x}\left(\theta \right)\pi \left(\theta \right)d\theta_{2}}{\int_{\Theta }L_{x}\left(\theta \right)\pi \left(\theta \right)d\theta }\frac{\int_{\Theta_{1}}L_{x}\left(\theta \right)\pi \left(\theta \right)d\theta_{1}}{\int_{\Theta }L_{x}\left(\theta \right)\pi \left(\theta \right)d\theta }\;. \end{array}$$

Due to the fact that $\theta_{1}⫫\theta_{2}$, it follows from the last equality in (A2) that

(A3) $$\begin{array}{cc} L_{x}\left(\theta \right)\pi_{1}\left(\theta_{1}\right)\pi_{2}\left(\theta_{2}\right)\;= & \;\pi_{1}\left(\theta_{1}\right)\int_{\Theta_{2}}L_{x}\left(\theta \right)\pi_{2}\left(\theta_{2}\right)d\theta_{2}\;\frac{\pi_{2}\left(\theta_{2}\right)\int_{\Theta_{1}}L_{x}\left(\theta \right)\pi_{1}\left(\theta_{1}\right)d\theta_{1}}{\int_{\Theta }L_{x}\left(\theta \right)\pi \left(\theta \right)d\theta }\;. \end{array}$$

The result follows considering, for instance,

(A4) $$L_{x}^{1}\left(\theta_{1}\right)=\int_{\Theta_{2}}L_{x}\left(\theta \right)\pi_{2}\left(\theta_{2}\right)d\theta_{2}\;\mathrm{and}\;L_{x}^{2}\left(\theta_{2}\right)=\frac{\int_{\Theta_{1}}L_{x}\left(\theta \right)\pi \left(\theta_{1}\right)d\theta_{1}}{\int_{\Theta }L_{x}\left(\theta \right)\pi \left(\theta \right)d\theta }.$$

□

Next, we prove the converse. Suppose that the likelihood can be factored as $L_{x}\left(\theta \right)=L_{x}^{1}\left(\theta_{1}\right)L_{x}^{2}\left(\theta_{2}\right)$. Then,

(A5) $$\begin{array}{c} \pi \left(\theta |x\right)=\frac{L_{x}\left(\theta \right)\pi \left(\theta \right)}{\int_{\Theta }L_{x}\left(\theta \right)\pi \left(\theta \right)d\theta }\;=\;\;\frac{L_{x}^{1}\left(\theta_{1}\right)L_{x}^{2}\left(\theta_{2}\right)\pi_{1}\left(\theta_{1}\right)\pi_{2}\left(\theta_{2}\right)}{\int_{\Theta_{1}\times \Theta_{2}}L_{x}^{1}\left(\theta_{1}\right)L_{x}^{2}\left(\theta_{2}\right)\pi_{1}\left(\theta_{1}\right)\pi_{2}\left(\theta_{2}\right)d\theta }. \end{array}$$

The posterior marginal density of $\theta_{i}$ is obtained from (A5) by integrating out the other component. Thus,

(A6) $$\begin{array}{c} \pi_{i}\left(\theta_{i}|x\right)\;=\;\frac{L_{x}^{i}\left(\theta_{i}\right)\pi_{i}\left(\theta_{i}\right)}{\int_{\Theta_{i}}L_{x}^{i}\left(\theta_{i}\right)\pi_{i}\left(\theta_{i}\right)d\theta_{i}}\;,\;i=1,2, \end{array}$$

and therefore, $\pi \left(\theta_{1},\theta_{2}|x\right)=\pi_{1}\left(\theta_{1}|x\right)\pi_{2}\left(\theta_{2}|x\right)$; that is, $\theta_{1}⫫\theta_{2}|X=x$. □

**Proof of Theorem** **3.** We first verify that $\theta_{2}$ is an NNP for testing $\bar{H}$ versus $\bar{A}$ by means of ${\bar{\varphi }}^{*}$. Recall that

(A7) $${\bar{\varphi }}^{*}\left(x,\theta_{2}\right)=1\Leftrightarrow \frac{{\bar{f}}_{\bar{H}}\left(x,\theta_{2}\right)}{{\bar{f}}_{\bar{A}}\left(x,\theta_{2}\right)}<\frac{b}{a}\;,$$

where ${\bar{f}}_{H}$ (${\bar{f}}_{A}$) is the predictive distribution for $\left(X,\theta_{2}\right)$ obtained under $\bar{H}$ ($\bar{A}$). In this case, the likelihood function generated by $\left(x_{0},\theta_{2}\right)$ for $\theta_{1}$ with $x_{0}\in X$ such that (1) holds and $\theta_{2}\in \Theta_{2}$ is

(A8) $$\begin{array}{cc} {\bar{L}}_{\left(x_{0},\theta_{2}\right)}\left(\theta_{1}\right)\;= & \;P\left(X=x_{0}\;|\;\theta_{1},\theta_{2}\right)\pi_{2}\left(\theta_{2}\;|\;\theta_{1}\right)\;=\;L_{x_{0}}\left(\theta_{1},\theta_{2}\right)\pi_{2}\left(\theta_{2}\right)\\ = & \;L_{x_{0}}^{1}\left(\theta_{1}\right)L_{x_{0}}^{2}\left(\theta_{2}\right)\pi_{2}\left(\theta_{2}\right). \end{array}$$

Then, the predictive function under the null hypothesis $\bar{H}$ can be calculated as

(A9) $$\begin{array}{c} {\bar{f}}_{\bar{H}}\left(x_{0},\theta_{2}\right)\;=\;\int_{\left\{\theta_{0}\right\}}{\bar{L}}_{\left(x_{0},\theta_{2}\right)}\left(\theta_{1}\right)dP_{\bar{H}}\left(\theta_{1}\right)\;, \end{array}$$

where $P_{\bar{H}}$ is degenerate at $\theta_{0}$ conditional distribution of $\theta_{1}|\theta_{1}=\theta_{0}$. Thus,

(A10) $${\bar{f}}_{\bar{H}}\left(x_{0},\theta_{2}\right)=L_{x_{0}}^{1}\left(\theta_{0}\right)L_{x_{0}}^{2}\left(\theta_{2}\right)\pi_{2}\left(\theta_{2}\right).$$

In addition, the predictive function under the alternative hypothesis $\bar{A}$ is given by

(A11) $$\begin{array}{cc} {\bar{f}}_{\bar{A}}\left(x_{0},\theta_{2}\right)\;= & \;\int_{\Theta_{1}}L_{x_{0}}^{1}\left(\theta_{1}\right)L_{x_{0}}^{2}\left(\theta_{2}\right)\pi_{2}\left(\theta_{2}\right)dP_{{\bar{H}}_{1}}\left(\theta_{1}\right)\\ = & \;\int_{\Theta_{1}}L_{x_{0}}^{1}\left(\theta_{1}\right)L_{x_{0}}^{2}\left(\theta_{2}\right)\pi_{2}\left(\theta_{2}\right)\pi_{1}\left(\theta_{1}\right)d\theta_{1}\\ = & \;L_{x_{0}}^{2}\left(\theta_{2}\right)\pi_{2}\left(\theta_{2}\right)\int_{\Theta_{1}}L_{x_{0}}^{1}\left(\theta_{1}\right)\pi_{1}\left(\theta_{1}\right)d\theta_{1}. \end{array}$$

Thus, the Bayes factor can be expressed by

(A12) $$\begin{array}{cc} \frac{{\bar{f}}_{\bar{H}}\left(x_{0},\theta_{2}\right)}{{\bar{f}}_{\bar{A}}\left(x_{0},\theta_{2}\right)}\;= & \;\frac{L_{x_{0}}^{1}\left(\theta_{0}\right)L_{x_{0}}^{2}\left(\theta_{2}\right)\pi_{2}\left(\theta_{2}\right)}{L_{x_{0}}^{2}\left(\theta_{2}\right)\pi_{2}\left(\theta_{2}\right)\int_{\Theta_{1}}L_{x_{0}}^{1}\left(\theta_{1}\right)\pi_{1}\left(\theta_{1}\right)d\theta_{1}}\\ = & \;\frac{L_{x_{0}}^{1}\left(\theta_{0}\right)}{\int_{\Theta_{1}}L_{x_{0}}^{1}\left(\theta_{1}\right)\pi_{1}\left(\theta_{1}\right)d\theta_{1}}. \end{array}$$

Note that Equation (A12) does not depend on $\theta_{2}$. As a result, the test in (A7) does not depend on $\theta_{2}$, and consequently, $\theta_{2}$ is an NNP for testing $\bar{H}$ versus $\bar{A}$ by means of ${\bar{\varphi }}^{*}$. Now, we shall determine the test $\varphi^{*}$ for *H* versus *A*. The predictive distribution for *X* at $x_{0}$ under the null hypothesis is

(A13) $$f_{H}\left(x_{0}\right)=\int_{\Theta_{1}\times \Theta_{2}}L_{x_{0}}\left(\theta_{1},\theta_{2}\right)dP_{H}\left(\theta_{1},\theta_{2}\right).$$

It is not difficult to verify that for fixed $\theta_{0}\in \Theta_{1}$, the conditional distribution of θ given $\theta_{1}=\theta_{0}$ is such that $\theta_{1}$ is degenerate at $\theta_{0}$, and $\theta_{2}$ is independent of $\theta_{1}$ with density $\pi_{2}$. Then,

(A14) $$\begin{array}{cc} f_{H}\left(x_{0}\right)\;= & \;\int_{\Theta_{1}\times \Theta_{2}}L_{x_{0}}\left(\theta_{1},\theta_{2}\right)dP_{H}\left(\theta_{1},\theta_{2}\right)\\ = & \;\int_{\Theta_{2}}L_{x_{0}}^{1}\left(\theta_{0}\right)L_{x_{0}}^{2}\left(\theta_{2}\right)\pi_{2}\left(\theta_{2}\right)d\theta_{2}\\ = & \;L_{x_{0}}^{1}\left(\theta_{0}\right)\int_{\Theta_{2}}L_{x_{0}}^{2}\left(\theta_{2}\right)\pi_{2}\left(\theta_{2}\right)d\theta_{2}. \end{array}$$

For the alternative hypothesis, we have that

(A15) $$\begin{array}{cc} f_{A}\left(x_{0}\right)\;= & \;\int_{\Theta_{1}\times \Theta_{2}}L_{x_{0}}^{1}\left(\theta_{1}\right)L_{x_{0}}^{2}\left(\theta_{2}\right)dP_{A}\left(\theta_{1},\theta_{2}\right)\\ = & \;\int_{\Theta_{1}\times \Theta_{2}}L_{x_{0}}^{1}\left(\theta_{1}\right)L_{x_{0}}^{2}\left(\theta_{2}\right)dP\left(\theta_{1},\theta_{2}\right)\\ = & \;\int_{\Theta_{1}}\int_{\Theta_{2}}L_{x_{0}}^{1}\left(\theta_{1}\right)L_{x_{0}}^{2}\left(\theta_{2}\right)\pi_{1}\left(\theta_{1}\right)\pi_{2}\left(\theta_{2}\right)d\theta_{1}d\theta_{2}\\ = & \;\int_{\Theta_{1}}L_{x_{0}}^{1}\left(\theta_{1}\right)\pi_{1}\left(\theta_{1}\right)d\theta_{1}\int_{\Theta_{2}}L_{x_{0}}^{2}\left(\theta_{2}\right)\pi_{2}\left(\theta_{2}\right)d\theta_{2}. \end{array}$$

Finally,

(A16) $$\begin{array}{cc} \frac{f_{H}\left(x_{0}\right)}{f_{A}\left(x_{0}\right)}\;= & \;\frac{L_{x_{0}}^{1}\left(\theta_{0}\right)\int_{\Theta_{2}}L_{x_{0}}^{2}\left(\theta_{2}\right)\pi_{2}\left(\theta_{2}\right)d\theta_{2}}{\int_{\Theta_{1}}L_{x_{0}}^{1}\left(\theta_{1}\right)\pi_{1}\left(\theta_{1}\right)d\theta_{1}\int_{\Theta_{2}}L_{x_{0}}^{2}\left(\theta_{2}\right)\pi_{2}\left(\theta_{2}\right)d\theta_{2}}\\ = & \;\frac{L_{x_{0}}^{1}\left(\theta_{0}\right)}{\int_{\Theta_{1}}L_{x_{0}}^{1}\left(\theta_{1}\right)\pi_{1}\left(\theta_{1}\right)d_{\theta_{1}}}\;=\;\frac{{\bar{f}}_{\bar{H}}\left(x_{0},\theta_{2}\right)}{{\bar{f}}_{\bar{A}}\left(x_{0},\theta_{2}\right)}\;. \end{array}$$

Hence,

(A17) $$\frac{f_{H_{0}}\left(x_{0}\right)}{f_{H_{1}}\left(x_{0}\right)}<\frac{a}{b}\Leftrightarrow \frac{{\bar{f}}_{{\bar{H}}_{0}}\left(x_{0},\theta_{2}\right)}{{\bar{f}}_{{\bar{H}}_{1}}\left(x_{0},\theta_{2}\right)}<\frac{a}{b},$$

and consequently,

(A18) $$\varphi^{*}\left(x_{0}\right)=1\Leftrightarrow {\bar{\varphi }}^{*}\left(x_{0},\theta_{2}\right)=1.$$

□

**Proof of Corollary** **1.** The corollary follows directly from Theorems 2 and 3. □

**Proof of Theorem** **4.** From Theorem 3 and Corollary 1, we have that for each $x_{0}\in X$,

(A19) $$\varphi^{*}\left(x_{0}\right)\;=\;1\Leftrightarrow \;BF\left(x_{0}\right)\;⩽\frac{b}{a}\;.$$

Then,

$$\varphi^{*}\left(x_{0}\right)\;=\;1\Rightarrow \;BF\left(x_{0}\right)\;⩽\;\frac{b}{a}\;\Rightarrow \;D\left(x_{0}\right)\;\subseteq \;D\;\Rightarrow$$

$$\Rightarrow \;\sum_{x\;\in \;D(x_{0})}f_{H,T(x_{0})}\left(x\right)\;\leq \;\sum_{x\;\in \;D}f_{H,T(x_{0})}\left(x\right)\;\Rightarrow \;p_{T}-value\left(x_{0}\right)\;\leq \;\alpha_{T}^{*}\left(x_{0}\right)\;.$$

Thus,

(A20) $$\varphi^{*}\left(x_{0}\right)\;=\;1\Rightarrow \;P_{T}-value\left(x_{0}\right)\;\leq \;\alpha_{T}^{*}\left(x_{0}\right)\;.$$

The converse is proven by the contrapositive.

$$\varphi^{*}\left(x_{0}\right)\;=\;0\Rightarrow \;\frac{b}{a}\;<\;BF\left(x_{0}\right)\;\Rightarrow \;D\;\cup \;\left\{x_{0}\right\}\;\subseteq \;D\left(x_{0}\right)\;.$$

As $x_{0}\notin D$ if $BF\left(x_{0}\right)>\frac{b}{a}$, we obtain that

$$\varphi^{*}\left(x_{0}\right)\;=\;0\;\Rightarrow \;\sum_{x\;\in \;D}f_{H,T(x_{0})}\left(x\right)\;+\;f_{H,T(x_{0})}\left(x_{0}\right)\;\leq \;\sum_{x\;\in \;D(x_{0})}f_{H,T(x_{0})}\left(x\right)\;.$$

Since $x_{0}\in D_{T}\left(x_{0}\right)$ and $BF\left(x_{0}\right)>\frac{b}{a}>0$, it follows that $f_{H,T(x_{0})}\left(x_{0}\right)>0$. Thus,

(A21) $$\varphi^{*}\left(x_{0}\right)\;=\;0\;\Rightarrow \;\alpha_{T}^{*}\left(x_{0}\right)\;<\;P_{T}-value\left(x_{0}\right)\;,$$

and consequently,

(A22) $$p_{T}-value\left(x_{0}\right)\;\leq \;\;\alpha_{T}^{*}\left(x_{0}\right)\;\Rightarrow \;\varphi^{*}\left(x_{0}\right)\;=\;1\;.$$

From (A20) and (A22), the result follows. □

**Mixed test for symmetry hypothesis for r×r contingency tables**

In this case, Table A1 represents the observed frequencies of the cross-classification of *n* units by the variables $X_{1}$ and $X_{2}$.

**Table A1:** Observed frequencies of $X_{1}$ and $X_{2}$ in the 3×3 case.

	$X_{2}$			
$X_{1}$	$x_{11}$	$x_{12}$	$x_{13}$	$n_{1.}$
	$x_{21}$	$x_{22}$	$x_{23}$	$n_{2.}$
	$x_{31}$	$x_{32}$	$x_{33}$	$n_{3.}$
	$n_{.1}$	$n_{.2}$	$n_{.3}$	*n*

Let $X=(X_{ij})$ be the $\left(r^{2}-1\right)$-dimensional vector of cell counts and $\theta =(\theta_{ij})$ be the $\left(r^{2}-1\right)$-dimensional vector of cell probabilities, where $X_{ij}$ and $\theta_{ij}$ are self explanatory. Suppose that *X* is a multinomial random vector with parameters *n* and θ. The likelihood function generated by x∈X for θ is given by

(A23) $$L_{x}\left(\theta \right)\;=\;\frac{n!}{x_{11}!\ldots x_{rr}!}\theta_{11}^{x_{11}}\ldots \theta_{rr}^{x_{rr}}.$$

The hypotheses for testing diagonal symmetry are

(A24) $$\begin{array}{cc} H: & \theta_{ij}=\theta_{ji}\;\forall i\neq j.\\ A: & \theta_{ij}\neq \theta_{ji}\;\mathrm{for}\;\mathrm{at}\;\mathrm{least}\;\mathrm{one}\;i\neq j. \end{array}$$

We also assume a prior Dirichlet distribution with parameter $\alpha =(\alpha_{ij})$ for θ. That is,

(A25) $$\pi \left(\theta \right)\;=\;\frac{\Gamma (\alpha_{11}+\ldots +\alpha_{rr})}{\Gamma \left(\alpha_{11}\right)\ldots \Gamma \left(\alpha_{rr}\right)}\theta_{11}^{\alpha_{11}-1}\ldots \theta_{rr}^{\alpha_{rr}-1}\;.$$

To perform the mixed test for the symmetry hypothesis, we consider the following reparametrization of the model: we define

(A26) $$\begin{array}{c} \lambda_{ij}\;=\;\frac{\theta_{ij}}{\theta_{ij}+\theta_{ji}}\;\;\;for\;i<j\\ \lambda_{ij}\;=\;\theta_{ij}+\theta_{ji}\;\;\;for\;i>j\\ \;\;\lambda_{ij}\;=\;\theta_{ij}\;for\;i=j. \end{array}$$

Let $\lambda =(\lambda_{1},\lambda_{2})$, where $\lambda_{1}$ is the $\left(\frac{r^{2}-r}{2}\right)$-dimensional vector for which the components are $\lambda_{ij}$’s such that i<j, and $\lambda_{2}$ is the $\left(\frac{r^{2}-r}{2}+r-1\right)$-dimensional vector for which the components are $\lambda_{ij}$’s such that i≥j. The new parameter space is then $\Lambda ={\left(0,1\right)}^{\frac{r^{2}-r}{2}}\times S_{\frac{r^{2}-r}{2}+r-1}$.

Then, we can rewrite the hypotheses (A24) as

(A27) $$\begin{array}{cc} \tilde{H}: & \lambda \in \Lambda_{0}\\ \tilde{A}: & \lambda \in \Lambda_{0}^{c}. \end{array}$$

where $\Lambda_{0}=B\times S_{\frac{r^{2}-r}{2}+r-1}$, and *B* is the singleton $B=\{\left(\frac{1}{2},\ldots ,\frac{1}{2}\right)\}$.

As in previous sections, we consider a statistic *T* that is s-ancillary for $\lambda_{1}$: *T* is the $\left(\frac{r^{2}-r}{2}+r-1\right)$-dimensional vector for which the components are the sums $X_{ij}+X_{ji}$ for i<j and $X_{ii}$ for i=1,…,r−1. The induced likelihood function for λ generated by *x* is

(A28) L˜x(λ)=∏i<jxij+xjixijλijxij(1−λij)xjin!∏ixii!∏i>j(xij+xji)!∏iλiixii∏i>jλijxij+xji.

We can easily see that the likelihood function in (A28) can be factored as ${\tilde{L}}_{x}\left(\lambda_{1},\lambda_{2}\right)={\tilde{L}}_{x}^{1}\left(\lambda_{1}\right){\tilde{L}}_{x}^{2}\left(\lambda_{2}\right)$. In addition, the prior distribution for λ is such that $\lambda_{1}$ and $\lambda_{2}$ are independent: $\lambda_{2}$ being a Dirichlet random vector and $\lambda_{1}$ a vector of independent Beta random variables. That is,

(A29) $$\tilde{\pi }\left(\lambda \right)\;=\;{\tilde{\pi }}_{1}\left(\lambda_{1}\right){\tilde{\pi }}_{2}\left(\lambda_{2}\right)\;=\;\prod_{i<j}\frac{\Gamma (\alpha_{ij}+\alpha_{ji})}{\Gamma \left(\alpha_{ij}\right)\Gamma \left(\alpha_{ji}\right)}\lambda_{ij}^{\alpha_{ij}-1}{\left(1-\lambda_{ij}\right)}^{\alpha_{ij}-1}I_{\left(0,1\right)}\left(\lambda_{ij}\right)\;{\tilde{\pi }}_{2}\left(\lambda_{2}\right)$$

From Theorem 3, we have that $\lambda_{2}$ is an NNP for testing $\tilde{H}$ versus $\tilde{A}$ by means of the mixed test. In addition, the mixed test for $\tilde{H}$ reduces to the mixed test for the simple hypothesis $\lambda_{1}=\left(\frac{1}{2},\ldots ,\frac{1}{2}\right)$ were $\lambda_{2}$ known. From Theorem 4, it follows that we only need to compare the conditional $P_{T}-value$ with the conditional adaptive significance level to test $\tilde{H}$ against $\tilde{A}$. From (28), (30) and (A28), we obtain for $x=(x_{ij})\in X$:

(A30) pT-valuex=∑y∈DT*(x)∏i<jyij+yjiyij(12)yij+yji=∑y∈DT*(x)∏i<jxij+xjiyij(12)xij+xji

and

(A31) αT*x=∑y∈D∩DT(x)∏i<jyij+yjiyij(12)yij+yji=∑y∈D∩DT(x)∏i<jxij+xjiyij(12)xij+xji.

In this case, these conditional quantities are simply determined by the products of binomial-type probabilities.
